# Brucellar, Pyogenic, and Tuberculous Spondylodiscitis at Tertiary Hospitals in Saudi Arabia: A Comparative Retrospective Cohort Study

**DOI:** 10.1093/ofid/ofad453

**Published:** 2023-08-28

**Authors:** Hajar AlQahtani, Fatimah Alzahrani, Ghaida Abalkhail, Hessa Bin Hithlayn, Husam I Ardah, Abdulrahman Alsaedy

**Affiliations:** Department of Pharmaceutical Care, Ministry of National Guard Health Affairs, Riyadh, Saudi Arabia; King Abdullah International Medical Research Center, Riyadh, Saudi Arabia; King Saud bin Abdulaziz University for Health Sciences, Riyadh, Saudi Arabia; Princess Nourah bint Abdulrahman University, Riyadh, Saudi Arabia; King Saud bin Abdulaziz University for Health Sciences, Riyadh, Saudi Arabia; King Saud University, Riyadh, Saudi Arabia; King Saud bin Abdulaziz University for Health Sciences, Riyadh, Saudi Arabia; Department of Biostatistics and Bioinformatics, King Abdullah International Medical Research Center, Riyadh, Saudi Arabia; King Abdullah International Medical Research Center, Riyadh, Saudi Arabia; Division of Infectious Diseases, Department of Medicine, King Abdulaziz Medical City, Ministry of National Guard Health Affairs, Riyadh, Saudi Arabia; College of Medicine, King Saud Bin Abdulaziz University for Health Sciences, Riyadh, Saudi Arabia

**Keywords:** infectious spondylodiscitis, microorganisms, surgical intervention

## Abstract

**Introduction:**

Spondylodiscitis is rare yet the most common form of spinal infection. It is characterized by inflammation of the intervertebral disk space and adjacent vertebral body. In Western countries, the incidence of spondylodiscitis is increasing. Clinical outcomes most commonly reported in the literature are the 1-year mortality rate (range, 6%–12%) and neurologic deficits.

**Methods:**

This multicenter retrospective cohort study assessed patients diagnosed with infectious spondylodiscitis who received treatment at King Abdulaziz Medical City in Riyadh and Jeddah, Saudi Arabia. All enrolled patients were ≥18 years old and were diagnosed per radiologic and microbiological findings and clinical manifestations between January 2017 and November 2021.

**Results:**

This study enrolled 76 patients with infectious spondylodiscitis, with a median age of 61 years. All patients presented with back pain for a median 30 days. Patients were stratified into 3 groups based on the causative pathogen: brucellar spondylodiscitis (n = 52), tuberculous spondylodiscitis (n = 13), and pyogenic spondylodiscitis (n = 11). All laboratory data and biochemical markers were not significantly different. However, C-reactive protein, erythrocyte sedimentation rate, and white blood cells were significantly different in the pyogenic spondylodiscitis group, with medians of 121 mg/dL (*P* = .03), 82 mmol/h (*P* = .04), and 11.2 × 109/L (*P* = .014), respectively.

**Conclusions:**

Back pain is a common clinical feature associated with infectious spondylodiscitis. The immense value of microbiological investigations accompanied with histologic studies in determining the causative pathogen cannot be emphasized enough. Treatment with prolonged intravenous antimicrobial therapy with surgical intervention in some cases produced a cure rate exceeding 60%.

Spondylodiscitis is rare yet the most common form of spinal infection. It is characterized by inflammation of the intervertebral disk space and adjacent vertebral body. It occurs via multiple mechanisms, including hematogenous seeding, where microorganisms travel from distant organs, and direct inoculation due to spinal surgery or trauma [1]. The predisposing factors include male sex, advanced age, diabetes mellitus, cancer, penetrating trauma, and spinal surgery [2]. In Western countries, the incidence of spondylodiscitis ranges from 0.4 to 2.4 per 100 000 individuals [3].

In a recent meta-analysis, 1756 cases of spondylodiscitis were analyzed: 56% of cases were male, the mean age was 60 years, and *Staphylococcus* spp and *Mycobacterium tuberculosis* were the most causative pathogens [4]. The lumbosacral region was the most affected area (∼50%), possibly because of the pump effect (high vasculature), and 30% of patients developed abscesses. Neurologic compromises were observed in 27.8% of the cases [4].

The clinical outcomes most commonly reported in the literature are the 1-year mortality rate (range, 6%–12%) and neurologic deficits [5–7]. Neurologic compromises that persist either improve or deteriorate in some patients after surgical or medical therapy, with a range of 5% to 26.7% depending on the treatment modality received, site/region of infection, association with abscess formation, and causative pathogens [5, 7–10]. The goal of this study was to characterize clinical, laboratory, and microbiological findings of cases with confirmed spontaneous spondylodiscitis and to evaluate outcomes after antimicrobial therapy.

## METHODS

### Study Setting

This multicenter retrospective cohort study assessed all patients diagnosed with infectious spondylodiscitis who received treatment at the King Abdulaziz Medical City (KAMC) in Riyadh and Jeddah, Saudi Arabia. KAMCs are accredited per the standards of Joint Commission International as 1500- and 600-bed tertiary care academic medical centers that provide care to Saudi National Guard soldiers, employees, and their families. As there was no hypothesis to be tested, we included all patients with infectious spondylodiscitis from January 2017 to November 2021 who met the inclusion criteria. Inclusion criteria were as follows: age ≥18 years; an admission diagnosis of spontaneous infectious spondylodiscitis; radiologic findings consistent with infectious spondylodiscitis per spine radiologists; clinical manifestations, including fever, back pain, and/or weight loss; microbiological findings, such as positive blood, tissue/bone, and abscess cultures or high titer in cases with brucellosis; and receipt of intravenous antimicrobial therapy for at least 6 weeks, with or without surgical intervention. Patients were excluded if they had life-threatening bacterial or fungal infections at the time of admission, required a prolonged hospital stay (>180 days), or had a penetrating spine injury or trauma.

The microbiological diagnosis of tuberculous spondylodiscitis was based on (1) the recovery of *M tuberculosis* from Lowenstein-Jensen agar and/or the presence of acid-fast bacilli in Ziehl-Neelsen–stained slides plus (2) caseation granulomatosis on histopathology with positive *M tuberculosis* polymerase chain reaction (PCR) test results from tissue, bone, or abscess specimens. The diagnosis of brucellar spondylodiscitis was based on clinical signs of brucellosis, *Brucella* spp growth in blood and other specimens, or serum brucella agglutination test results ≥1:320. To diagnose spontaneous spondylodiscitis caused by bacteria other than *M tuberculosis* or *Brucella* spp, bone aspiration specimens obtained through needle biopsy under computed tomography in sterile and bedside conditions were sent to the microbiology laboratory for bacterial culture. Once bacterial growth was observed in conventional cultures, automated systems and conventional methods were used for bacterial identification and antimicrobial susceptibilities. Primary outcomes include mortality at 90 and 365 days, clinical cure, and staging of neurologic compromises as compared with baseline at the end of antimicrobial therapy. Secondary outcomes were recurrence rate of spondylodiscitis within 3 months after completion of antimicrobial therapy and adverse drug reactions resulting from antimicrobial therapy.

### Definitions

**Clinical Cure.** Clinical cure is defined as follows:

- Resolution of biochemical signs of infection: normalization of white blood cells, C-reactive protein (CRP), and erythrocyte sedimentation rate (ESR)
- Radiologic improvement of affected disk/vertebrae: regression or stabilization of destruction associated with spine infection when compared with baseline imaging
- Resolution of back pain or improvement of neurologic deficit based on Dr Darouiche’s staging scoring system [11]

**Neurologic Staging.** Neurologic staging was developed to evaluate the progression of symptoms associated with infection and physical findings at baseline assessment:

Stage 1: back pain at the level of the affected spine
Stage 2: nerve root pain radiating from the involved spinal area
Stage 3: motor weakness, sensory deficit, and bladder and bowel dysfunction
Stage 4: paralysis [11].

This system was used in this study during baseline assessment and diagnosis of infectious spondylodiscitis and at the end of antimicrobial therapy.

### Statistical Analysis

The study participants were divided into 3 groups based on the type of spondylodiscitis: brucellar, tuberculosis, and pyogenic. Categorical data were presented as frequencies and percentages. Continuous variables were presented by the normality of their distribution—mean ± SD for normally distributed data or median (IQR) for nonnormally distributed data—overall and in groups of spondylodiscitis types. The 3 groups were compared with a χ^2^ test or Fisher exact test for categorical variables. Analysis of variance and the Kruskal-Wallis test were used for continuous variables, as appropriate. The level of significance was set at *P* = .05. Statistical analyses were conducted with SAS version 9.4 (SAS Institute Inc).

## RESULTS

A total of 156 patients were admitted to KAMC with a diagnosis of infectious spondylodiscitis. Of these, 40 had culture-negative infectious spondylodiscitis, 10 had confirmed central nervous system involvement, and 30 were lost to follow-up at 3 months and were thus excluded. Seventy-six patients remained, who were predominantly male (67.1%) and had a median age of 61 years. Diabetes mellitus and hypertension were the most frequently observed comorbidities. All patients presented with back pain for a median 30 days (IQR, 14–120). However, only 29 (38.2%) had a fever before presentation. All laboratory data and biochemical markers were not significantly high except for CRP, ESR, and white blood cell, with medians of 121 mg/dL (IQR, 35–208; *P* = .03), 82 mmol/h (IQR, 57–107; *P* = .04), and 11.2 × 10^9^/L (IQR, 6–16; *P* = .014), respectively. Further details are listed in Table 1. The patients were stratified into 3 groups based on the causative pathogen: brucellar spondylodiscitis (G1, n = 52), tuberculous spondylodiscitis (G2, n = 13), and pyogenic spondylodiscitis (G3, n = 11).

**Table 1.** Baseline Characteristics of 76 Patients With Infectious Spondylodiscitis

	Spondylodiscitis, Median (IQR) or No. (%)				
	G1: Brucellar (n = 52)	G2: Tuberculous (n = 13)	G3: Pyogenic (n = 11)	Total (N = 76)	*P* Value
Demographics					
Age, y	61 (53–71)	42 (32–78)	62 (52–80)	61 (49–73)	.79
Female	16 (30.8)	4 (30.8)	5 (45.5)	25 (32.9)	.66
Medical history: baseline comorbidities					
Diabetes mellitus	24 (46.2)	1 (7.7)	6 (54.5)	31 (40.8)	.025
Hypertension	23 (44.2)	2 (15.4)	6 (54.5)	31 (40.8)	.10
Liver disease	3 (5.8)	1 (7.7)	3 (27.3)	7 (9.2)	.08
Chronic kidney disease	5 (9.6)	1 (7.7)	2 (18.2)	8 (10.5)	.73
Congestive heart failure	0	1 (7.7)	2 (18.2)	3 (3.9)	.017
Malignancy	2 (3.8)	1 (7.7)	0	3 (3.9)	.69
History of active tuberculosis	5 (9.6)	7 (53.8)	0	12 (15.8)	.005
History of brucellosis	42 (80.8)	0	3 (27.3)	45 (59.2)	<.001

### G1: Brucellar Spondylodiscitis

Fifty-two patients were diagnosed with brucellar spondylodiscitis and were predominantly male (n = 36, 70%) with a median age of 61 years. The most common comorbidity in this group was a history of brucellosis in 42 (80.8%), followed by diabetes (46.2%) and hypertension (44.2%). Although only 20 (38.5%) had a fever, backache was consistent in all patients, occurring for 14 to 120 days before admission. Most patients evaluated for neurologic symptoms had stage 1 pain, which manifested as back pain only at the level of the affected disk space (n = 28, 53.8%), followed by stage 2, which is pain radiating from the affected spinal area (n = 8, 15.4%), and stage 3, weakness and neurologic deficit (n = 16, 30.8%); none of the patients reached stage 4, paralysis. Most patients had a history of ingesting unpasteurized dairy products and/or having animal contact.

Microbiological diagnostic investigations revealed that 29 (55.8%) patients had positive cultures: 24 and 5 had positive blood and tissue/bone cultures, respectively. The diagnosis for the rest was made with brucella titers; 23 patients had high brucella titers, with medians of 640 and 1280 for *Brucella abortus* and *Brucella melitensis* antibodies (Table 2). The lumbar spine region was the most affected for most patients (n = 41, 79%), followed by the thoracic and cervical areas. Most patients had at least 2 vertebrae infected. Further details are presented in Table 3. Eleven (21.2%) patients underwent surgical intervention, of whom 10 had spinal abscesses. Data on the types of surgical intervention and abscesses are shown in Table 3. All patients received doxycycline as the backbone of their antimicrobial therapy in combination with different agents, of which 10, 33, and 9 patients received dual, triple, and quadruple therapies. The median duration of antimicrobial therapy was 173 days (IQR, 96–255). The mortality rate was zero in the brucellar spondylodiscitis group at 90 and 365 days. The clinical cure rate was 66% (33/50) in patients followed up until the end of antimicrobial therapy (2 patients were lost to follow-up at the end of therapy). Most (80%) patients were pain-free at the end of their antimicrobial therapy; however, 9 still had back pain by the end of therapy. Three patients experienced a recurrence of brucellar spondylodiscitis within 3 months of completing treatment for the primary infection. Nineteen patients developed adverse drug reactions to therapy, including acute kidney injury (n = 2, 3.80%), gastrointestinal upset (n = 9, 17.3%), elevated liver enzymes (n = 2, 3.8%), myelosuppression (n = 2, 3.8%), and allergic reactions (n = 3, 5.8%; Table 4).

**Table 2.** Clinical and Microbiological Features of 76 Patients With Infectious Spondylodiscitis

	Spondylodiscitis, Median (IQR) or No. (%)				
	G1: Brucellar (n = 52)	G2: Tuberculous (n = 13)	G3: Pyogenic (n = 11)	Total (N = 76)	*P* Value
Initial presentation					
Presence of back pain	52 (100)	13 (100)	11 (100)	76 (100)	
Duration of back pain, d	30 (14–120)	120 (30–240)	30 (10–90)	30 (14–120)	.13
Fever	20 (38.5)	3 (23.1)	6 (54.5)	29 (38.2)	.26
Laboratory findings^a^					
CRP, mg/L	32 (12.3–69)	17 (13.9–43)	121 (35.6–208)	34.6 (14–75)	.03
ESR, mm/h	59 (39–82)	48 (38–57)	82 (5–107)	57.5 (41–82)	.04
WBCs, 10^9^/L	6.3 (5.3–8)	8.2 (6–10)	11.2 (6–16.6)	6.7 (5.6–9.6)	.01
Lactic acid, mmol/L	1.1 (0.78–1.66)	0.9 (0.91–0.91)	2.5 (1.5–19.9)	1.5 (0.9–2.3)	.07
Procalcitonin, ng/mL	0.1 (0.03–0.48)	0.1 (0.04–0.09)	0.2 (0.17–0.3)	0.2 (0.03–0.3)	.5
Serum creatine, mmol/L	67 (60–87)	62 (48–77)	64.5 (62–116)	65.5 (60–87)	.3
Neurologic staging at initial presentation					.4
Stage 1	28 (53.8)	6 (46.2)	6 (54.5)	40 (52.6)	
Stage 2	8 (15.4)	2 (15.4)	2 (18.2)	12 (15.8)	
Stage 3	16 (30.8)	4 (30.8)	2 (18.2)	22 (28.9)	
Stage 4	0	1 (7.7)	1 (9.1)	2 (2.6)	
Source of infection					
Contact with animals	8 (15)	…	2 (18.2)	10 (13.2)	<.001
Ingestion of unpasteurized dairy products	44 (85)	…	…	43 (56.6)	<.001
Endocarditis	…	…	3 (27.3)	3 (3.9)	<.001
Skin/bone infections	…	…	2 (18.2)	2 (2.6)	<.001
Pulmonary tuberculosis	…	13 (100)	…	14 (18.4)	<.001
Unknown	…	…	4 (36.4)	4 (5.3)	<.001
Microbiological findings					
Conventional culture methods					
Positive blood culture	24 (46.2)	0	6 (54.5)	32 (42)	.09
Positive tissue/bone culture^b^	5 (9.6)	9 (69.2)	5 (45.5)	19 (25)	<.001
Positive deep wound culture	0	0	2 (18.2)	2 (2.6)	.07
Additional serologic and molecular testing					
*Brucella abortus* antibody	640 (320–1280)	…	…	…	…
*Brucella melitensis* antibody	1280 (320–5120)	…	…	…	…
*Mycobacterium tuberculosis*^c^	…	13 (100)	…	13 (17)	…

Abbreviations: CRP, C-reactive protein; ESR, erythrocyte sedimentation rate; PCR, polymerase chain reaction; WBC, white blood cell.

a On the day when infectious spondylodiscitis diagnosis was confirmed.

b Computed tomography–guided biopsy culture or surgical biopsy.

c Positive PCR for *M tuberculosis* from biopsy specimen.

**Table 3.** Distribution of Involved Vertebral Regions, Surgical Interventions, and Presence of Abscess per Group

	Spondylodiscitis, No. (%)				
	G1: Brucellar (n = 52)	G2: Tuberculous (n = 13)	G3: Pyogenic (n = 11)	Total (N = 76)	*P* Value
Involved vertebral regions					
Cervical	7 (13.5)	2 (15.4)	…	9 (11.8)	.56
Thoracic	8 (15.4)	4 (30.8)	1 (9.1)	13 (17)	.37
Lumbar	27 (52)	5 (38.5)	6 (54.5)	38 (50)	.65
Thoracic-lumbar	4 (7.7)	1 (7.7)	3 (27.3)	8 (10.5)	.13
Cervical-lumbar	5 (9.6)	…	…	5 (6.6)	.48
Cervical-thoracic	1 (1.9)	…	2 (18.2)	3 (3.9)	.07
Sacral-lumbar	5 (9.6)	…	…	5 (6.6)	.48
No. of affected vertebrae					.26
1	9 (17.3)	1 (7.7)	…	10 (13.2)	
2	36 (69)	10 (77)	8 (72.7)	49 (64.5)	
3	5 (9.6)	1 (7.7)	1 (9.1)	6 (7.9)	
4	1 (1.9)	1 (7.7)	…	2 (2.6)	
≥5	1 (1.9)	…	2 (18.2)	2 (2.6)	
Surgical intervention for presence of abscess	11 (21.2)	5 (38.5)	1 (9.1)	17 (22.4)	.2
Types of surgery					
Discectomy and fusion	3 (6)	1 (8)	…	4 (5)	.6
Laminectomy and fusion	1 (1.9)	…	…	1 (1)	
Corpectomy with instrumentation and decompression	1 (1.9)	…	…	1 (1)	
Decompression	1 (1.9)	3 (23)	…	4 (5)	
Decompression and fusion	1 (1.9)	…	…	1 (1)	
Decompression and fixation	…	1 (8)	…	1 (1)	
Fixation of spine	4 (8)	…	1 (9)	5 (6.6)	
Presence of abscess					
Psoas abscess	1 (1.9)	3 (23.1)	2 (18.2)	6 (7.9)	.01
Paraspinal abscess	4 (7.7)	5 (38.5)	4 (36.4)	13 (17.1)	.04
Epidural abscess	5 (9.6)	1 (7.7)	1 (9.1)	7 (9.2)	>.99

**Table 4.** Outcomes of Interest

	Spondylodiscitis, No. (%)				
	G1: Brucellar (n = 52)	G2: Tuberculous (n = 13)	G3: Pyogenic (n = 11)	Total (N = 76)	*P* Value
Primary outcome					
Mortality at day 90	0	0	2 (18.2)	2 (2.6)	.02
Clinical cure at end of antimicrobial therapy	33 (66)	8 (72.7)	7 (63.6)	48 (66.7)	>.99
Neurologic staging after antimicrobial therapy					.7
Stage 0	39 (85)	9 (90)	6 (75)	54 (79)	
Stage 1	11 (24)	1 (10)	2 (25)	14 (21)	
Secondary outcome					
Recurrence of spondylodiscitis within 3 mo after completing treatment of primary infection	3 (6)	…	1 (9.1)	4 (5.3)	.57
Development of acute kidney injury related to antimicrobial therapy	2 (3.8)	…	1 (9.1)	3 (3.9)	.44
Development of gastrointestinal symptoms related to antimicrobial therapy	9 (17.3)	1 (7.7)	1 (9.1)	11 (14.5)	.7
Development of myelosuppression related to antimicrobial therapy	2 (3.8)	…	1 (9.1)	3 (3.9)	.44
Elevation in liver enzymes	2 (3.8)	…	2 (18.2)	4 (5.3)	.15
Development of allergic reaction	3 (5.8)	…	…	3 (3.9)	>.99
Development of any adverse drug reaction related to antimicrobial therapy	18 (34.6)	1 (7.7)	5 (45.5)	24 (31.6)	.3

### G2: Tuberculous Spondylodiscitis

Patients in G2 were younger than those in G1 or G3, with a median age of 42 years. The patients were predominantly male (69.8%), and half had a history of active tuberculosis. The duration of pain was longer, at a median 30 days, but it could extend to 240 days. The distribution of the initial staging used to evaluate neurologic deficits was as follows: stage 1 (n = 6, 46.2%), 2 (n = 2, 15.4%), 3 (n = 4, 30.8%), and 4 (n = 1, 7.7%). The diagnosis was confirmed by positive cultures and PCR; in 9 (69.2%) patients, *M tuberculosis* grew from tissue or bone cultures, and all patients had positive PCR results from biopsy specimens. The thoracic and lumbar areas were the most affected, and at least 2 vertebrae were infected (Table 3). Nine patients had spinal abscesses, of which 5 required surgical intervention after drainage. Most patients received first-line quadruple therapy, including rifampin, isoniazid, pyrazinamide, ethambutol, and moxifloxacin, in different combinations for *M tuberculosis*. The median treatment duration was 300 days (IQR, 270–455; Table 5). The primary outcomes were clinical cure 72.7% (8/11; where 2 patients were lost to follow-up appointments by the end of their treatment), no recurrence within 90 days, and no mortality at 90 or 365 days posttreatment. Further details are presented in Table 5.

**Table 5.** Antimicrobial Treatment Regimens According to Causative Organism and Susceptibility

Treatment Regimen: Duration of Therapy, Median (IQR)
Pyogenic spondylodiscitis: 120 d (76–365)
MSSA (n = 3)
Cefazolin switched to clindamycin (IV then PO)
Cefazolin switched to clindamycin PO + Augmentin PO
Cefazolin switched to ceftriaxone then clindamycin PO
MRSA (n = 3)
Trimethoprim/sulfamethoxazole + rifampin
Vancomycin switched to rifampin + trimethoprim/sulfamethoxazole
*Clostridium sordellii* (n = 1)
Augmentin PO
Viridans group *Streptococcus* (n = 2)
Ceftriaxone
Ceftriaxone switched to cephalexin
*Klebsiella pneumoniae* (n = 1)
Ertapenem
*Enterococcus faecalis* (n = 1)
Piperacillin/tazobactam switched to Augmentin
Brucellar spondylodiscitis: 173 d (96–255)
Dual therapy (n = 10)
Doxycycline + rifampicin
Doxycycline + ciprofloxacin
Doxycycline + streptomycin
Doxycycline + trimethoprim/sulfamethoxazole
Rifampin + ciprofloxacin
Rifampin + trimethoprim/sulfamethoxazole
Triple therapy (n = 33)
Doxycycline + streptomycin + rifampin
Doxycycline + streptomycin + ciprofloxacin
Doxycycline + clindamycin + ciprofloxacin.
Doxycycline + trimethoprim/sulfamethoxazole + rifampin
Doxycycline + gentamycin + rifampin
Doxycycline + ciprofloxacin + gentamycin
Doxycycline + ciprofloxacin + rifampicin
Doxycycline + ciprofloxacin + trimethoprim/sulfamethoxazole
Quadruple therapy (n = 9)
Doxycycline + ciprofloxacin + rifampin + trimethoprim/sulfamethoxazole
Doxycycline + ciprofloxacin + rifampin + streptomycin
Doxycycline + ciprofloxacin + rifampin + gentamycin
Doxycycline + streptomycin switched to gentamycin + rifampin
Tuberculous spondylodiscitis: 300 d (270–455)
Dual therapy (n = 1)
Rifampin + isoniazid
Quadruple therapy (n = 12)
Rifampin + pyrazinamide + moxifloxacin + ethambutol
Rifampin + isoniazid + pyrazinamide + ethambutol
Rifampin + isoniazid + moxifloxacin + ethambutol

Abbreviations: IV, intravenous; MRSA, methicillin-resistant *Staphylococcus aureus*; MSSA, methicillin-resistant *Staphylococcus aureus*; PO, by mouth.

### G3: Pyogenic Spondylodiscitis

Patients were older in G3 with a median age of 62 years, and the majority were male (54.5%). More comorbidities were reported in G3: diabetes and hypertension (54.5%), liver disease (27.3%), chronic kidney disease (18.2%), and congestive heart failure (18.2%). The initial presentation of pyogenic infectious spondylodiscitis was as follows: 11 (100%) patients had pain for a median 30 days (IQR, 10–90), and 6 (54.5%) had a fever. The initial stages for neurologic deficit assessment were stage 1 (n = 6, 54.5%), 2 (n = 2, 18.2%), 3 (n = 2, 18.2%), and 4 (n = 1, 9.1%). The sources of infection were endocarditis (n = 3, 27.3%) and skin and bone infections (n = 2, 18.2%); 4 patients had unknown sources of infection (36.4%; Table 2). The diagnosis was confirmed by blood, tissue/bone, and wound cultures. Six (54.5%), 5 (45.5%), and 2 (18.2%) patients had positive blood, tissue/bone, and deep wound cultures, respectively. Most patients experienced infection in the lumbar region with at least 2 vertebrae impacted (n = 9, 81.8%). Only 1 patient required surgical intervention, for which internal fixation of the spine was performed. Seven patients developed abscesses in the epidural, paraspinal, and psoas regions. The antimicrobial therapy varied depending on the causative organism, with a median treatment duration of 120 days. Further details are presented in Table 5. Two patients died within 90 days of infectious spondylodiscitis diagnosis. The clinical cure rate was 63.6%, and 1 (9.1%) patient had a recurrence of infectious spondylodiscitis within 90 days of completing therapy for the primary infection. Five patients developed adverse drug reactions to the therapy, including elevated liver enzyme levels (n = 2, 18.2%), acute kidney injury (n = 1, 9.1%), gastrointestinal upset (n = 1, 9.1%), and myelosuppression (n = 1, 9.1%; Table 4).

## DISCUSSION

Infectious spondylodiscitis is a rare spinal infection with a low incidence [3]. Recently, many studies have indicated an increased number of reported cases of infectious spondylodiscitis, up to 2.4 per 100 000 cases in Western countries [3]. Local data are lacking on the incidence of infectious spondylodiscitis in patients with spinal infections.

Our study focused on evaluating all infectious spondylodiscitis cases in 2 large academic teaching centers in Saudi Arabia. Saudi Arabia is endemic for tuberculosis and brucellosis; unsurprisingly, the combinations accounted for >50% of the cases. In this study, we retrospectively followed patients with spontaneous infectious spondylodiscitis of different etiologies for 1 year; to our knowledge, this study is the first of its kind. Of the 76 patients, 52 (68.4%), 13 (17%), and 11 (14.5%) had brucellar, tuberculous, and pyogenic spondylodiscitis, respectively. Data from Turkey, southern Spain, and Italy have reported that the rate of brucellosis spondylodiscitis is between 20% and 50% [12, 13]. Close contact with livestock and ingesting unpasteurized dairy products, a common occurrence in some geographic areas among the Saudi population, are probable causes.

The rate of tuberculous spondylodiscitis declined at our institution when compared with the results of Al Othman et al (13 cases over 5 years vs 69 cases over 15 years) [14]. In contrast, a prospective study conducted in Egypt noted that in the last 5 years, higher incidences of tuberculosis were reported among its communities (15/44) [10]. *Staphylococcus aureus* was the most frequently isolated pathogen in patients with pyogenic spondylodiscitis, with a methicillin-resistant *S aureus* rate of 50%. *S aureus* was the most common organism associated with pyogenic spondylodiscitis, in >50% of cases with positive cultures in many previous studies [6, 7, 15].

The median age of patients in the current study was 61 years (IQR, 49–73), consistent with previous studies in which spondylodiscitis was reported more frequently in elderly patients [16, 17]. In terms of each group, patients in the tuberculous spondylodiscitis group were younger, with a median age of 42 years (IQR, 32–78), as compared with the other groups in this study and in different national and international studies, with a mean age of 50 years [14, 18]. Diabetes mellitus and hypertension were the most frequently observed comorbidities (41%), which is consistent with the results of other studies [7, 8, 15]. Therefore, diabetes is considered the leading risk factor for infectious spondylodiscitis. More than 50% of our cohort had a history of active tuberculosis or brucellosis, indicating the endemicity of both diseases in the Arabian Peninsula.

Infectious spondylodiscitis is suspected when clinical symptoms are present, such as fever, localized back pain, and spinal deformity [10]. Back pain was reported in all cases, whereas fever was noted in approximately 40% of patients in our study, as opposed to 54% by Waheed et al and Turunc et al [10, 12]. High-grade fever was more strongly associated with pyogenic spondylodiscitis (6/11 in the current study) than with tuberculous and brucellar spondylodiscitis. This finding can be explained by the correlation between the duration of pain and the presence of fever, since 75% of the brucellar and tuberculous groups had pain for 120 and 240 days, respectively, indicating that spondylodiscitis was a late complication of tuberculosis and brucellosis. In contrast, Turunc et al found that most of their patients had spondylodiscitis early in the disease course [12]. A unique evaluation of pain and accompanying neurologic manifestations was performed with Dr Darouiche's grading system and compared with other studies [11]. Neurologic symptoms were most frequently reported, at approximately 50% in each group. Neurologic deficits were more common in the brucellar and tuberculous groups, with more spinal cord compression and disc/bone destruction caused by granuloma formation [12, 19]. Regarding spinal anatomic site involvement, the lumbar region appeared to be the most affected area, secondary to increased vasculature and proximity of infection, especially in brucellar spondylodiscitis [7, 10, 12, 13]. Most patients had at least 2 vertebral bodies involved (n = 49), 10 of whom required surgery. The decision to perform spinal surgical intervention was individualized depending on the extent of disc/bone destruction, abscess formation, and the effect on the patient's mobility. We also considered patient-specific parameters such as age and comorbidities. As surgical intervention serves as prompt source control, 17 patients underwent it with antimicrobial therapy, mostly for brucellar spondylodiscitis (n = 11). The timeline of surgical intervention ranges between the first week of hospital admission (n = 13) and 3 to 12 weeks (n = 4) after starting antimicrobial therapy.

In contrast to findings from Turunc et al and Waheed et al, inflammatory markers, including CRP and ESR, were significantly higher in the pyogenic group, which we believe is related to the disease being an early vs late complication of the primary infection [10, 12]. However, our results are consistent with those of Colmenero et al, where the pyogenic group had higher CRP and ESR levels and more leukocytosis [13].

Intravenous antimicrobial therapy is the cornerstone of infectious spondylodiscitis treatment, in addition to surgical intervention in some cases [3]. It is worth mentioning that antimicrobials have a variable ability to penetrate bone tissue, ranging from moderate with β-lactams and glycopeptides to good or excellent with sulfamethoxazole-trimethoprim, fluoroquinolones, and rifampin [3]. Combination therapy is indicated in tuberculous and brucellar spondylodiscitis secondary to the nature of the disease, which has been proven to reduce relapse episodes, lower the risk of resistance, and shorten the duration of therapy [20, 21]. More than 80% of patients in the brucellar spondylodiscitis group received ≥3 active agents, which may have contributed to the lower relapse rate in our cohort than in other studies [20]. The brucellar spondylodiscitis group had a longer duration of therapy, at a median 173 days (IQR, 96–255), than the pyogenic group, which is consistent with the literature [17, 19]. In contrast to brucellar spondylodiscitis, the rules of combination therapy are not well established for pyogenic spondylodiscitis treatment [22].

Regarding the duration of antimicrobial therapy, it seems that patients with pyogenic spondylodiscitis required a shorter duration, at a median 120 days, as compared with brucellar and tuberculous spondylodiscitis (180 and 300 days, respectively). These findings are consistent with those of expert recommendations and previous studies [5, 7, 14]. Moreover, recommendations endorsed by international guidelines for treatment of spine infections include a minimum duration of 6, 12, and up to 54 weeks for pyogenic, brucellar, and tuberculous spine infections, respectively [23, 24].

Indeed, 22.4% of the patients in our study underwent surgery, with a higher number for brucellar spondylodiscitis, unlike the study by Turunc et al, who reported a higher rate of tuberculous spondylodiscitis requiring surgical intervention as compared with brucellar and pyogenic spondylodiscitis [12].

In our study, 26 patients developed abscesses at various locations: 17%, 9%, and 7% had paraspinal, epidural, and psoas abscesses, respectively. Conversely, according to Titlic and Josipovic-Jelic and Kaya et al, spondylodiscitis is often associated with psoas and epidural abscesses [25, 26].

The overall clinical cure rate was >60% (48/72), consistent with the report of Waheed et al [10]. The 90-day mortality was 2.6%, close to that by Waheed et al (4.5%) and Colmenero et al (3.2%) [10, 13]. However, Karadimas et al cited higher mortality rates, which may be related to the nature of the cases, mainly pyogenic spondylodiscitis [7].

Four patients had a recurrence of spondylodiscitis within 3 months of completing therapy, similar to that by Colmenero et al [13]. In brucellar spondylodiscitis, 3 patients had a recurrence, 2 received dual therapy, and 1 received a short therapy duration of 70 days. The last patient with recurrence had methicillin-resistant *S aureus* spondylodiscitis and required dual therapy with trimethoprim-sulfamethoxazole and rifampin for 1 year.

Throughout the therapy period, side effects from antibiotics occurred in 31.6% of patients in our study, higher than that by Aagaard et al (23%) [6]. The most common side effect reported by Aagaard et al was rash (n = 11) [6]. In contrast, gastrointestinal upset was the predominant side effect (n = 11) in this study.

Some limitations of this study should be considered when interpreting our findings. Considering its retrospective nature, this study is at a high risk of selection and sampling biases. However, we attempted to minimize this by applying stringent inclusion criteria. The study was not sufficiently powered to detect significant differences among the groups owing to the small sample size. Finally, some patients (2 patients in G1 and G2) missed follow-up for outcome evaluation and were excluded from the final analysis.

## CONCLUSION

Treatment with prolonged courses of antibiotics with or without surgical intervention resulted in a cure rate exceeding 60%.

## Notes

***Author contributions.*** H. A. designed the study methodology. F. A, G. A., and H. B. H. collected the data. H. A., F. A., G. A., H. H., H. I. A., and A. A. analyzed the data and reviewed the initial draft of the manuscript. All authors agreed on final version of the manuscript for final submission.

***Data availability*.** Data sets are available upon reasonable request from the first author (H. A.; Hajar.alqahtani99@gmail.com).

***Patient consent statement.*** The design of this study was approved by King Abdullah International Medical Research Center’s Institutional Review Board (RSS22R/014/07) and conforms to standards currently applied in the Kingdom of Saudi Arabia. The center approved a waiver of consent since the study does not include factors necessitating patient consent.

***Financial support.*** H. A. acknowledges academic support from the College of Pharmacies at King Saud University, Princess Nourah bint Abdulrahman University, King Saud Bin Abdulaziz University for Health Sciences, and King Abdullah International Medical Research Center.
